# Neratinib stimulates senescence of mammary cancer cells by reducing the levels of SIRT1

**DOI:** 10.18632/aging.205882

**Published:** 2024-05-31

**Authors:** Wenhuan Li, Peng Fu, Pengfei Shi, Bo Hu, Hai Li

**Affiliations:** 1 Department of Thyroid and Breast Surgery, The Central Hospital of Wuhan, Tongji Medical College of Huazhong University of Science and Technology, Wuhan 430030, Hubei, China

**Keywords:** neratinib, breast cancer, mammary cancer cells, cellular senescence, SIRT1

## Abstract

Neratinib, a typical small-molecule, pan-human tyrosine kinase inhibitor (TKI), has been licensed for the treatment of human epidermal growth factor receptor 2 (HER2)-positive breast cancer. However, the underlying pharmacological mechanism is still unknown. In the current study, we report a novel function of Neratinib by showing that its treatment stimulates senescence of the mammary cancer AU565 cells. Our results demonstrate that Neratinib induces mitochondrial injury by increasing mitochondrial reactive oxygen species (ROS) and reducing intracellular adenosine triphosphate (ATP). Also, we found that Neratinib induced DNA damage by increasing the levels of 8-Hydroxy-desoxyguanosine (8-OHdG) and γH2AX in AU565 cells. Additionally, Neratinib reduced the levels of telomerase activity after 7 and 14 days incubation. Importantly, the senescence-associated-β-galactosidase (SA-β-Gal) assay revealed that Neratinib stimulated senescence of AU565 cells. Neratinib decreased the gene levels of human telomerase reverse transcriptase (hTERT) but increased those of telomeric repeat-binding factor 2 (TERF2) in AU565 cells. Further study displayed that Neratinib upregulated the expression of K382 acetylation of p53 (ac-K382) and p21 but reduced the levels of sirtuin-1 (SIRT1). However, overexpression of SIRT1 abolished the effects of Neratinib in cellular senescence. These findings provide strong preclinical evidence of Neratinib’s treatment of breast cancer.

## INTRODUCTION

Breast cancer has emerged as the first high-incidence cancer worldwide. Its incidence is growing at a rate of about 0.5% per year [1]. According to data released by the American Cancer Society in 2023, it is expected that there will be 300,590 new cases of breast cancer and 43,700 deaths from breast cancer in the United States in 2023 [2]. In 2022, American women were diagnosed with 287,850 new cases of invasive breast cancer and 51,400 cases of ductal carcinoma *in situ*, resulting in 43,250 deaths from breast cancer [3]. The incidence of breast cancer is influenced by various factors including genetic predisposition, environmental factors, age, and lifestyle choices [4]. The main cause of death in breast cancer patients is cancer recurrence and metastasis [5]. Although the overall survival rate for breast cancer patients can reach 90% in five years, there is a high risk of recurrence and metastasis during treatment, leading to tumor deterioration and even death. Furthermore, adverse reactions such as hair loss, gastrointestinal reactions, cardiac toxicity, bone marrow suppression, and drug resistance resulting from radiotherapy and chemotherapy pose serious challenges to the treatment of breast cancer patients. Therefore, it is urgent to find new effective and low-toxic chemotherapy drugs and targeted drugs [1]. Based on molecular typing, breast cancer is divided into four subtypes, among which human epidermal growth factor receptor 2 (HER2)-positive breast cancer accounts for about 20%. HER2-positive breast cancer has biological characteristics of easy recurrence and poor prognosis, which is caused by HER2 gene amplification and overexpression that can accelerate the growth of cancer cells and increase their invasive and metastatic abilities [6]. Therefore, the serious clinical consequences of HER2-positive breast cancer and the economic burden it brings to the world urgently require effective treatment.

Cellular senescence was first described by Hayflick and Moorhead in 1961 and can be caused by various stresses such as physical and chemical damage, telomere shortening, activation of oncogenes, as well as DNA damage due to factors such as ionizing radiation and chemotherapy drugs [7]. Exposure to chemotherapeutic agents can lead to two outcomes for both normal and tumor cells: apoptosis-programmed cell death and therapy-induced cellular senescence (TIS), a permanent arrest of cell proliferation leading to premature aging [8]. It is reported that cisplatin can upregulate p53 and p21, inducing HepG2 cells to undergo senescence [9]. Inducing tumor cell senescence can inhibit tumor cell proliferation and invasion [10], and has been shown to effectively inhibit tumor proliferation in clinical cancer treatment [11, 12]. In breast cancer research, promising anti-tumor effects have also been achieved by inducing cell senescence [13].

Neratinib was first approved for extended adjuvant treatment of HER-2-positive breast cancer in the United States in 2017 [14]. Neratinib can reversibly bind to the ATP binding site in the tyrosine kinase region of HER2 through hydrogen bonding, preventing ATP binding and inhibiting tyrosine kinase phosphorylation and activation to inhibit cell proliferation [15]. It has been shown that HER-2 inhibitors exert a significant inducing effect on tumor cell senescence [16]. However, whether Neratinib exerts its anti-tumor activity through inducing breast cancer cell senescence is not clear. Herein, the regulatory effect of Neratinib on AU565 cell senescence and its potential mechanism were explored to provide a solid theoretical basis for the clinical use of Neratinib in treating breast cancer.

## RESULTS

### Neratinib induced cytotoxicity in mammary cancer AU565 cells

**Figure 1:**
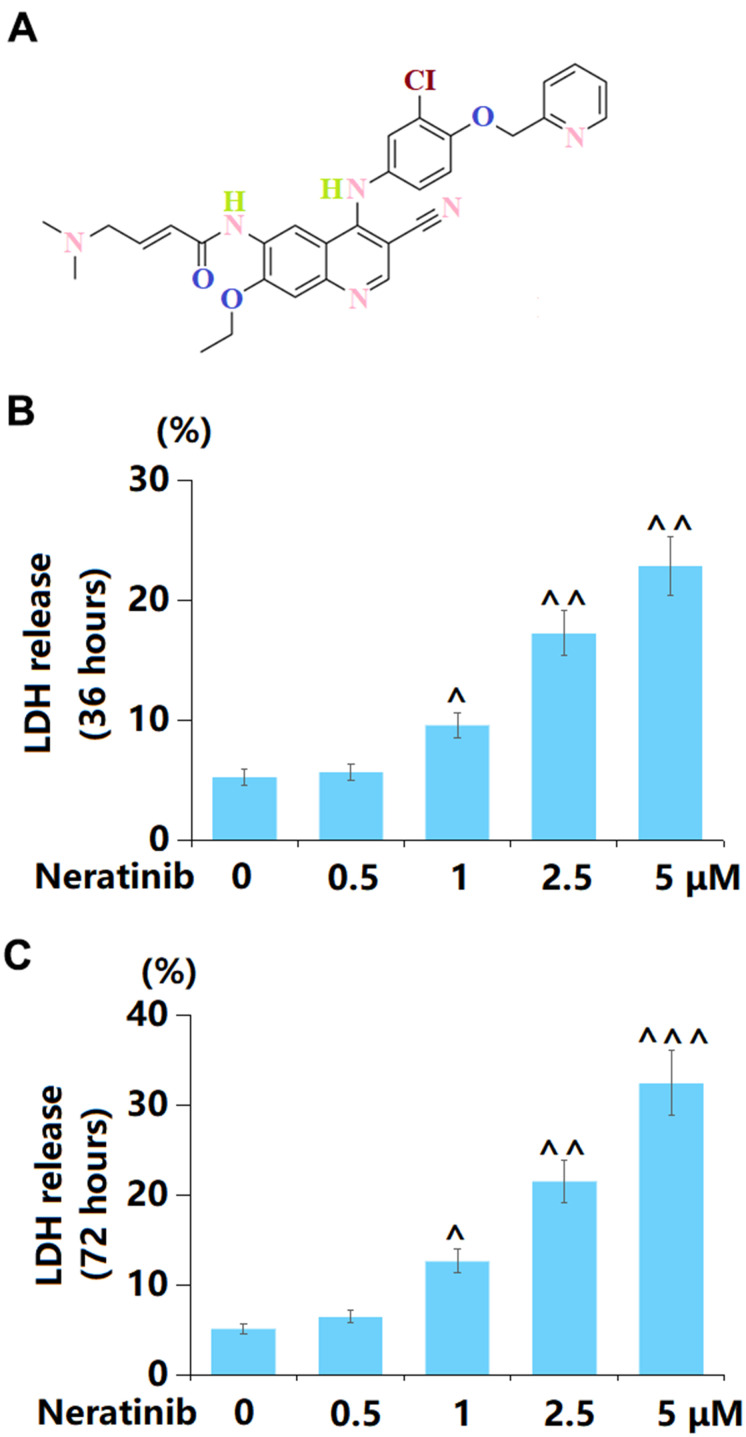
**Neratinib induced cytotoxicity in mammary cancer AU565 cells.** (**A**) Molecular structure of Neratinib; (**B**) Cells were stimulated with 0.5, 1, 2.5, 5 μM Neratinib for 36 hours. LDH release was measured using a commercial kit; (**C**) Cells were stimulated with 0.5, 1, 2.5, and 5 μM Neratinib for 72 hours. LDH release was measured using a commercial kit (^, ^^, ^^^, P<0.05, 0.01, 0.005 vs. vehicle group).

The molecular structure of Neratinib is illustrated in Figure 1A. To assess the toxic effect of Neratinib on AU565 cells, cells were stimulated with 0.25, 0.5, 1, 2.5, and 5 μM Neratinib for 36 h and 72 h, respectively. After 36 h incubation, the LDH release was maintained at around 5% under the stimulation of 0.25 and 0.5 μM Neratinib but sharply increased to 9.6%, 17.3%, and 22.9% by 1, 2.5, and 5 μM Neratinib, respectively (Figure 1B). Moreover, after 72 h incubation, the LDH release in 0.25, 0.5, 1, 2.5, and 5 μM Neratinib-treated AU565 cells was 5.2%, 6.5%, 12.7%, 21.6%, and 32.5%, respectively (Figure 1C). Thus, 1, 2.5, and 5 μM Neratinib were selected for subsequent experiments.

### Neratinib induced the production of mitochondrial ROS and mitochondrial dysfunction

**Figure 2:**
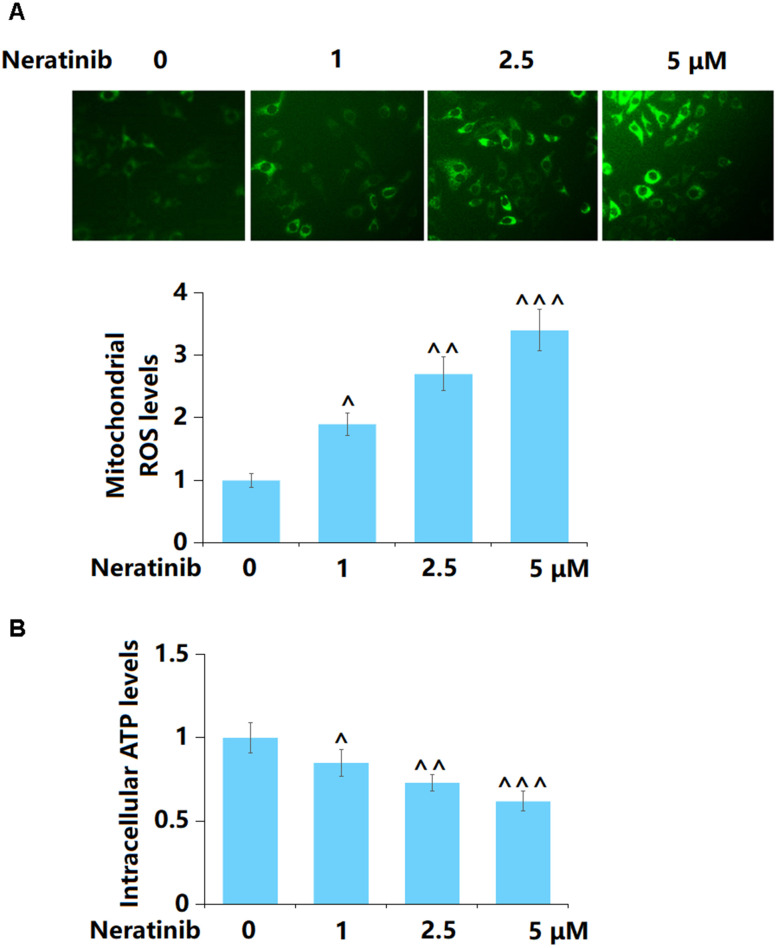
**Neratinib induced the production of mitochondrial ROS and mitochondrial dysfunction.** AU565 cells were stimulated with Neratinib (1, 2.5, 5 μM) for 36 hours. (**A**) The levels of mitochondrial ROS were measured using Mitosox Green; (**B**) The levels of intracellular ATP (^, ^^, ^^^, P<0.05, 0.01, 0.005 vs. vehicle group).

AU565 cells were stimulated with Neratinib (1, 2.5, and 5 μM) for 36 h. The mitochondrial ROS level was sharply increased by 1, 2.5, and 5 μM Neratinib (Figure 2A). Furthermore, the level of intracellular ATP in AU565 cells was markedly reduced by 1, 2.5, and 5 μM Neratinib (Figure 2B).

### The effects of Neratinib on DNA damage in AU565 cells

**Figure 3:**
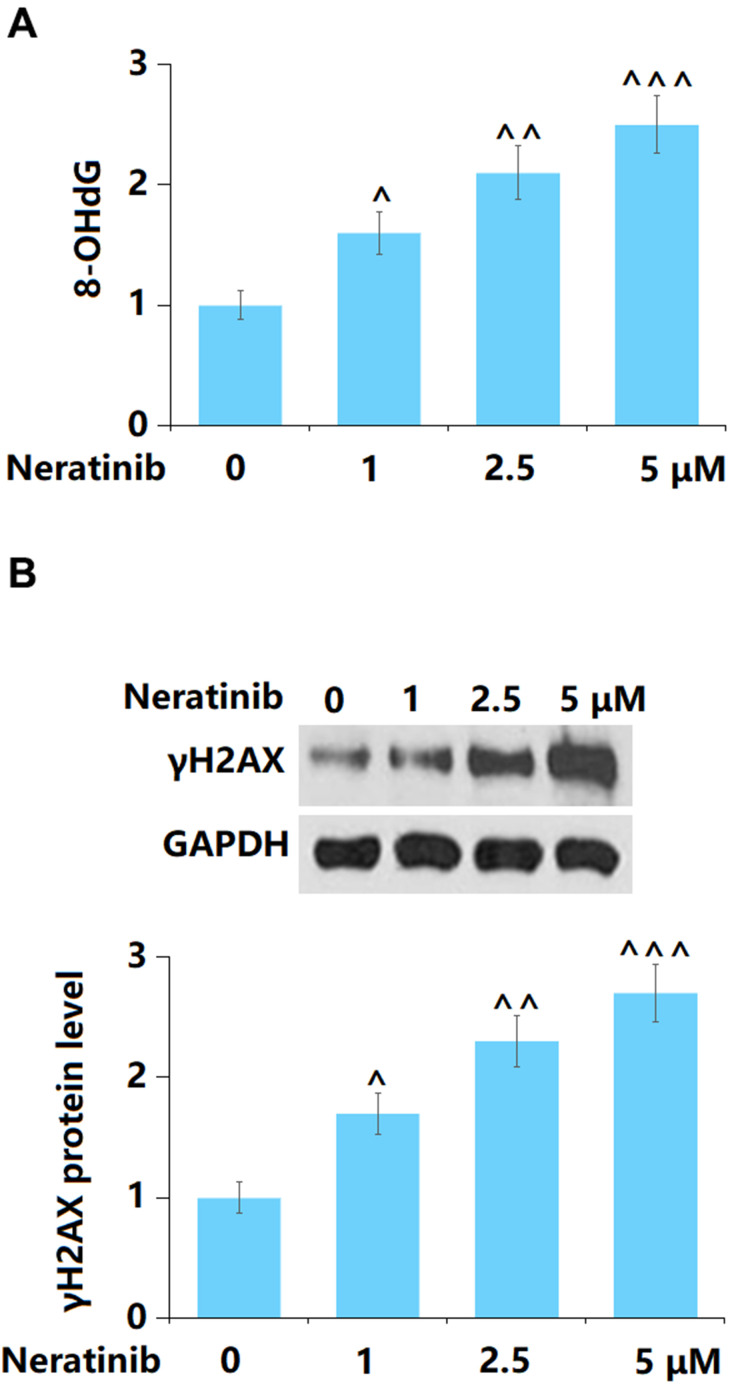
**The effects of Neratinib in DNA damage in AU565 cells.** Cells were stimulated with Neratinib (1, 2.5, 5 μM) for 72 hours. (**A**) The levels of 8-OHdG as measured by a commercial kit; (**B**) The levels of γH2AX (^, ^^, ^^^, P<0.05, 0.01, 0.005 vs. vehicle group).

8-OHdG and γH2AX are critical biomarkers of DNA damage [17]. AU565 cells were stimulated with Neratinib (1, 2.5, and 5 μM) for 72 h. The 8-OHdG (Figure 3A) and γH2AX (Figure 3B) levels in AU565 cells were remarkably elevated as the concentration of Neratinib increased from 1 to 5 μM, implying a facilitating effect of Neratinib on DNA damage in AU565 cells.

### Neratinib reduced the levels of telomerase activity in AU565 cells

**Figure 4:**
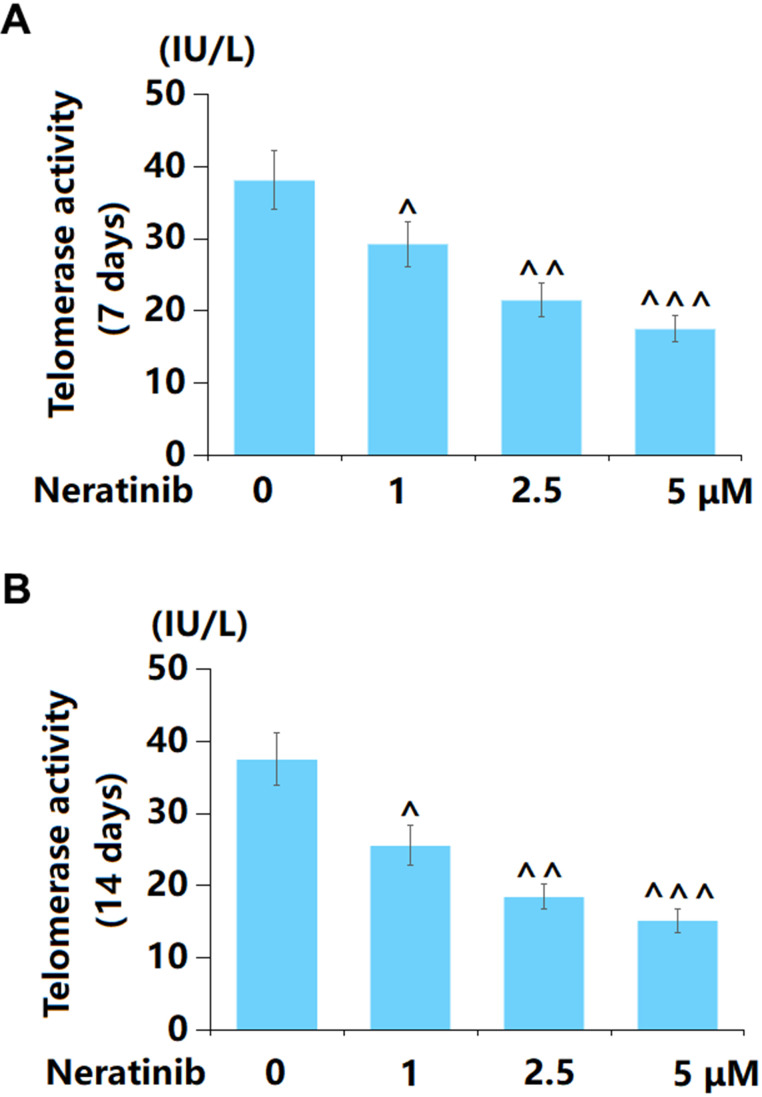
**Neratinib reduced the levels of telomerase activity in AU565 cells.** (**A**) Cells were stimulated with Neratinib (1, 2.5, 5 μM) for 7 days. The telomerase activity was assayed; (**B**) Cells were stimulated with Neratinib (1, 2.5, 5 μM) for 14 days. The telomerase activity was assayed (^, ^^, ^^^, P<0.05, 0.01, 0.005 vs. vehicle group).

The declined activity of telomerase is a crucial sign of cell senescence [18]. AU565 cells were stimulated with 1, 2.5, and 5 μM Neratinib for 7 and 14 days. After 7-day incubation, the telomerase activity in AU565 cells was markedly declined from 38.1 to 29.3, 21.5, and 17.5 IU/L by 1, 2.5, and 5 μM Neratinib, respectively (Figure 4A). After 14-day incubation, the telomerase activity in AU565 cells was notably reduced from 37.5 to 25.6, 18.5, and 15.2 IU/L by 1, 2.5, and 5 μM Neratinib, respectively (Figure 4B).

### Neratinib stimulated cellular senescence in AU565 cells

**Figure 5:**
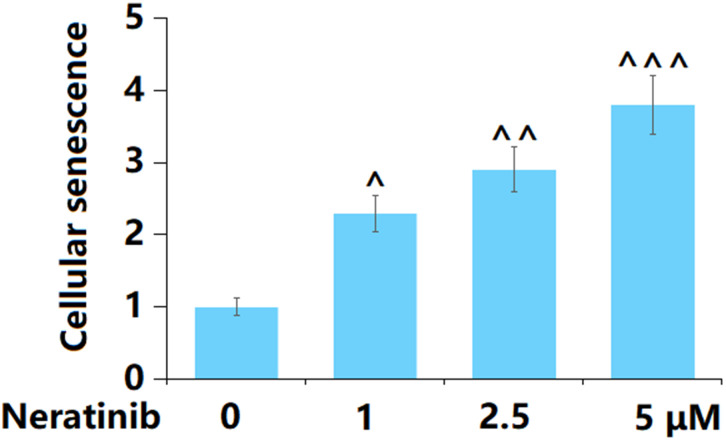
**Neratinib stimulated cellular senescence in AU565 cells.** Cells were stimulated with Neratinib (1, 2.5, 5 μM) for 14 days. The SA-β-Gal assay was used to assess cellular senescence (^, ^^, ^^^, P<0.05, 0.01, 0.005 vs. vehicle group).

AU565 cells were stimulated with Neratinib (1, 2.5, and 5 μM) for 14 days, after which cellular senescence was determined. The proportion of SA-β-Gal positive cells was notably elevated by 1, 2.5, and 5 μM Neratinib, suggesting a facilitating effect of Neratinib on the cellular senescence in AU565 cells (Figure 5).

### Neratinib decreased the gene levels of hTERT but increased the gene levels of TERF2 in AU565 cells

**Figure 6:**
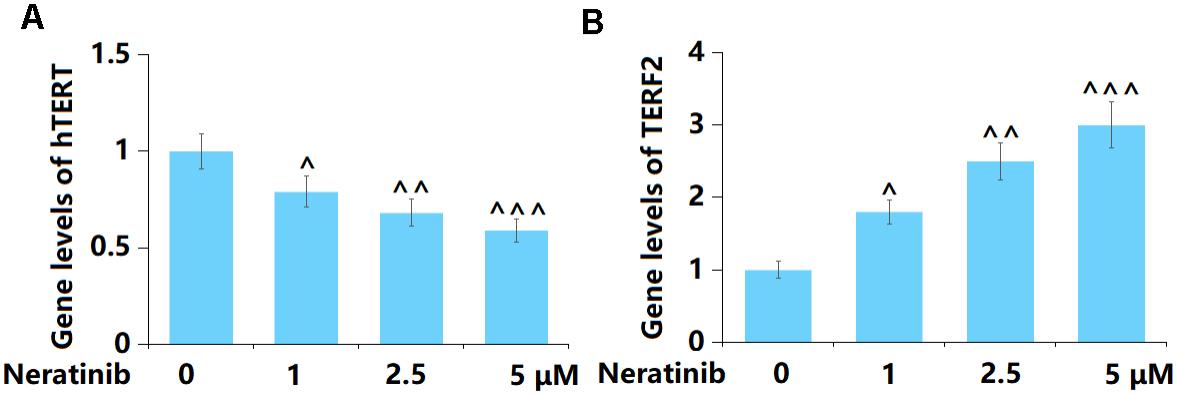
**Neratinib decreased the gene levels of hTERT but increased the gene levels of TERF2 in AU565 cells.** Cells were stimulated with Neratinib (1, 2.5, 5 μM) for 14 days. (**A**) Gene levels of hTERT; (**B**) Gene levels of TERF2 (^, ^^, ^^^, P<0.05, 0.01, 0.005 vs. vehicle group).

To further confirm the regulatory effect of Neratinib on cell senescence in AU565 cells, the levels of hTERT and TERF2 were determined. The hTERT level was notably reduced (Figure 6A), while that of TERF2 was markedly increased (Figure 6B) by 1, 2.5, and 5 μM Neratinib.

### The effects of Neratinib in the expression of K382 acetylation of p53 (ac-K382) and p21

**Figure 7:**
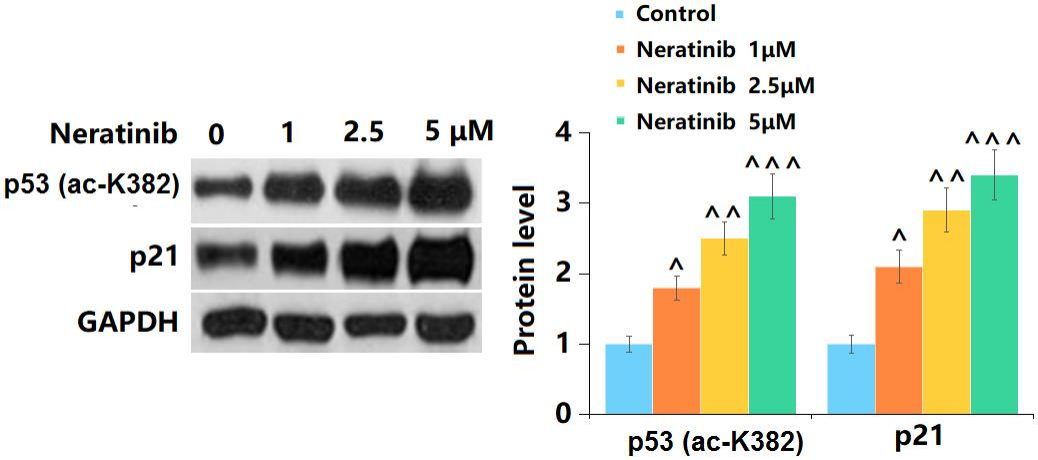
**The effects of Neratinib in the expression of K382 acetylation of p53 (ac-K382) and p21.** (**A**) Representative images of p53 (ac-K382) and p21 protein as measured by Western blot analysis (^, ^^, ^^^, P<0.05, 0.01, 0.005 vs. vehicle group).

p53 and p21 are vital signals involved in cell senescence [19]. Herein, p53 (Figure 7A) and p21 (Figure 7B) were significantly upregulated by 1, 2.5, and 5 μM Neratinib after 14 days incubation. This further confirmed the effects of Neratinib on cell senescence.

### The effects of Neratinib in the expression of SIRT1

**Figure 8:**
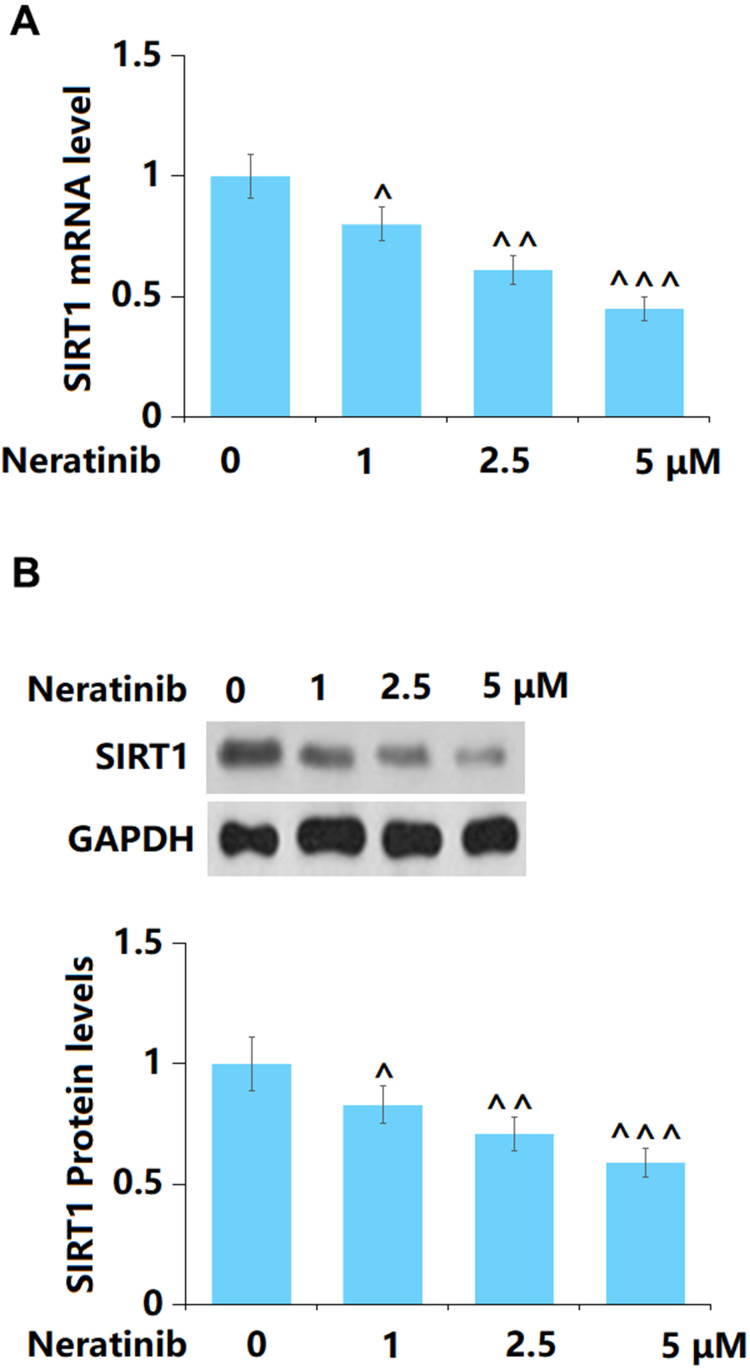
**The effects of Neratinib in the expression of SIRT1.** (**A**) mRNA of SIRT1; (**B**) Protein levels of SIRT1 as measured by Western blot (^, ^^, ^^^, P<0.05, 0.01, 0.005 vs. vehicle group).

SIRT1 has recently been reported to participate in the processing of cell senescence [20]. Herein, both the gene (Figure 8A) and protein levels (Figure 8B) of SIRT1 were found to be remarkably reduced by 1, 2.5, and 5 μM Neratinib after 14 days incubation.

### Overexpression of SIRT1 abolished the effects of Neratinib in cellular senescence

**Figure 9:**
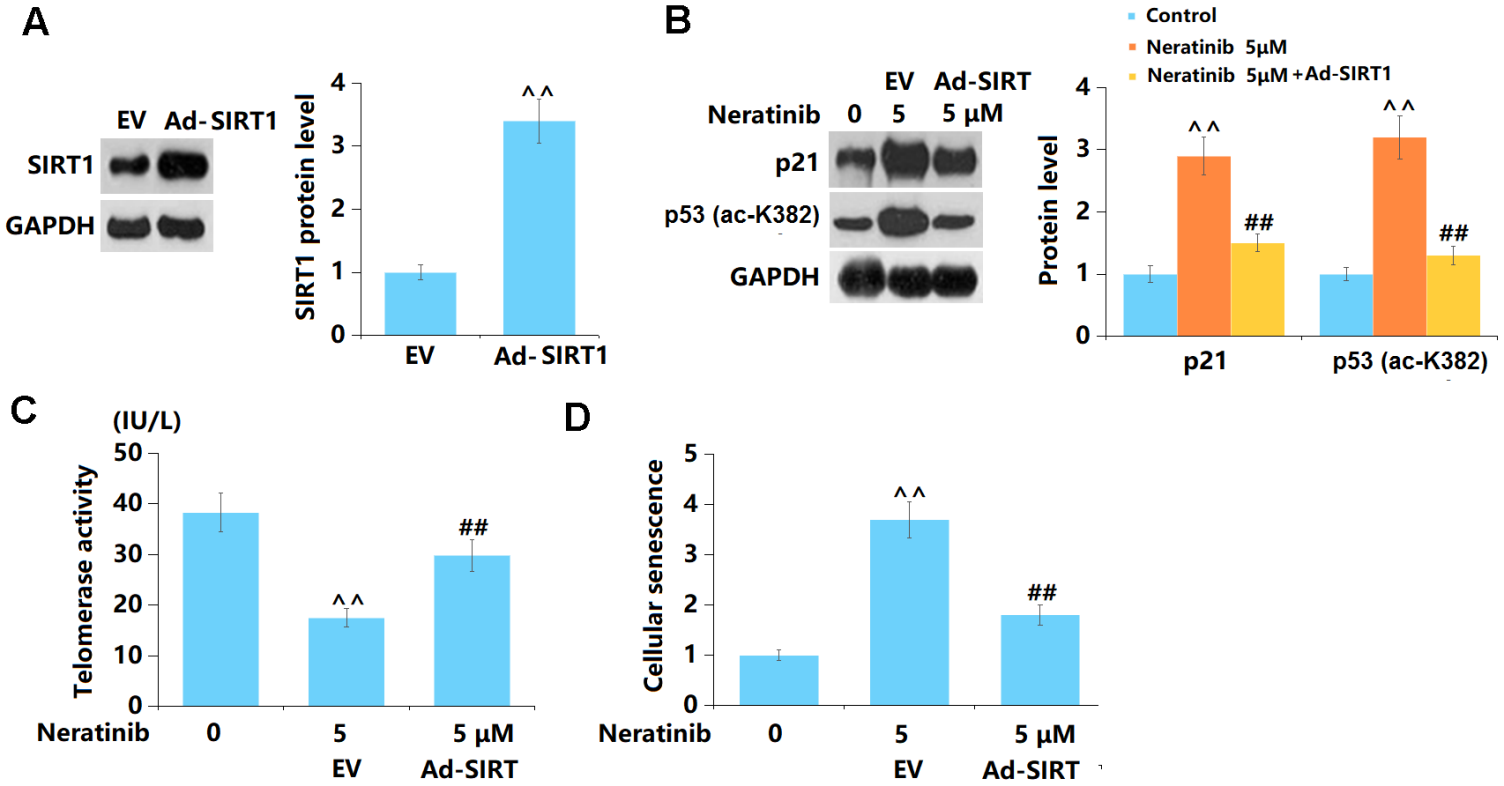
**Overexpression of SIRT1 abolished the effects of Neratinib in cellular senescence.** Cells were transduced with Ad-SIRT1, followed by stimulation with Neratinib (5 μM). (**A**) Western blot results revealed successful overexpression of SIRT1; (**B**) Representative images of p53 (ac-K382) and p21; (**C**) The telomerase activity; (**D**) The SA-β-Gal assay was used to assess cellular senescence (^^, P<0.01 vs. vehicle group; ##, P<0.01 vs. Neratinib group).

To elucidate the role of SIRT1 in Neratinib-induced cell senescence in AU565 cells, cells were transduced with Ad-SIRT1 and subsequently stimulated with Neratinib (5 μM). The overexpression of SIRT1 in AU565 cells was verified using the Western blot assay (Figure 9A). Levels of p53 and p21 were sharply increased by Neratinib and notably reduced by overexpressing SIRT1 (Figure 9B). In addition, the telomerase activity was reduced from 38.3 to 17.5 in Neratinib-stimulated AU565 cells and notably increased to 29.8 by overexpressing SIRT1 (Figure 9C). Furthermore, the increased proportion of SA-β-Gal positive cells induced by Neratinib was markedly repressed by overexpressing SIRT1 (Figure 9D).

## DISCUSSION

Significant changes in cell morphology and structure occur during senescence, including increases in cell and nuclear size, flattening and irregularity, vacuole formation, abnormal nuclear membrane, accumulation of lysosomes and mitochondria, as well as chromatin condensation and distribution changes [21]. In addition, senescent cells exhibit higher levels of lysosomes and β-galactosidase activity, which can be specifically detected using the substrate analog X-Gal. Increased SA-β-Gal activity under pH6.0 conditions is a key feature of senescent cells and is currently the most commonly used biomarker in senescence research [22]. In recent years, decreased telomerase activity has been used in combination with or as an alternative to the SA-β-Gal method for detecting cell senescence [23, 24]. Common nuclear biomarkers include senescence-associated DNA damage foci (SDFs), activation of ATM kinase and its phosphorylated substrate γ-H2AX, senescence-associated heterochromatic foci (SAHF) characterized by increased methylation of histone H3K9, as well as proteins such as heterochromatin protein 1 (HP1), high mobility group A (HMG-A), and macroH2A [25, 26]. Herein, the cytotoxic function of Neratinib in AU565 cells was accompanied by facilitated cell senescence and declined telomerase activity, implying that the anti-tumor property of Neratinib in breast cancer might be correlated to the induction of cell senescence. Moreover, the enhanced cell senescence in AU565 cells induced by Neratinib was accompanied by increased production of mitochondrial ROS and mitochondrial dysfunction, similar to the effect of artesunate in colorectal cancer cells reported by Huang [27]. Furthermore, the DNA damage in AU565 cells was markedly induced by Neratinib, which further confirmed its facilitating effect on the cell senescence in mammary cancer cells.

Human telomerase reverse transcriptase (hTERT) plays a crucial role in cell senescence. During cell division, the telomeres at the end of chromosomes become gradually shortened. When the telomeres shorten to a certain degree, the cell enters the senescence process. The hTERT enzyme encoded by the hTERT gene can reverse such process by synthesizing new DNA sequences at the telomere ends, thereby extending the telomere length and allowing the cell to continue dividing and maintain a youthful state [28, 29]. Telomeric repeat-binding factor 2 (TERF2) is a protein that binds to telomeric DNA and forms a protective complex called the telomere complex, which induces the development of cell senescence [30, 31]. Herein, hTERT was markedly downregulated and TERF2 markedly upregulated by Neratinib in AU565 cells, indicating an inducing effect of Neratinib on cell senescence in mammary cancer cells by impacting the telomerase activity.

After cells are stimulated by external stimuli, p53 is activated, initiating the expression of relevant cyclin-dependent kinase inhibitors (CDKI), such as p21^Waf1/Cip1^, thereby inhibiting the phosphorylation of Rb and stopping cell proliferation to induce cell senescence [32, 33]. In the absence of p53, the abnormal increase of p21 in normal embryonic cells can induce cell senescence [34]. In non-small cell lung cancer cells, when p53 is missing, the activation of protein kinase C (PKC) by the activator phorbol 12-myristate 13-acetate (PMA) leads to the up-regulation of p21 expression, resulting in G2/M phase arrest and the appearance of senescent phenotypes such as increased cell volume and β-galactosidase activity [35]. Herein, the Neratinib-induced cell senescence in AU565 cells was accompanied by an upregulation of p21 and p53, in line with the senescence-inducing function of Resveratrol in mammary cancer cells reported by Ma [36]. SIRT1 is a multifunctional transcription regulator with NAD-dependent protein deacetylase activity. When SIRT1 is activated, the cell lifespan can be extended by affecting processes such as cell cycle and cell death, thus combating cell senescence [37]. In addition, SIRT1 inhibits the activity of p53 and p21 by reducing their acetylation levels, thereby preventing premature cell senescence or death [38, 39]. Herein, in line with the function of SIRT1 in the senescence of liver cancer cells reported by Wang [40], the anti-tumor and senescence-facilitating function of Neratinib in AU565 cells were accompanied by sharply downregulated SIRT1. Moreover, the influence of Neratinib on the cell senescence in AU565 cells was rescued by overexpressing SIRT1, implying that Neratinib might induce the cell senescence in mammary cancer cells by downregulating SIRT1. In our future work, the anti-tumor property and senescence-inducing function of Neratinib will be further identified using an *in situ* xenograft model.

In conclusion, our findings demonstrate that Neratinib promotes cell senescence of mammary cancer cells by inducing DNA damage and reducing telomerase activity mediated by inhibition of SIRT1. These findings reveal novel insight into the underlying mechanism whereby Neratinib exerts its anti-cancer actions in mammary cancer, which broadens our understanding of its anti-cancer property.

## MATERIALS AND METHODS

### Cells and transfection

AU565 cells were obtained from iCELL (China) and cultured in a specific medium for AU565 cells (iCell-h468-001b, iCELL, China) at 37° C with 5% CO_2_. To achieve SIRT1-ovexpressed AU565 cells, cells were transduced with Ad-SIRT1 for 2 days, the efficacy of which was identified using the Western blot assay.

### Lactate dehydrogenase (LDH) release

After centrifuging cells, 150 μL of LDH release reagent was added. The culture plate was gently shaken and then incubated for 1 hour. The supernatant was removed and 60 μL of LDH detection working solution was introduced, followed by placement in the dark for 30 minutes. The absorbance at 450 nm was measured to calculate the LDH release.

### The detection of the mitochondrial ROS

MitoSOX Green (#M36008, Thermo Fisher Scientific, USA) was diluted to 5mM with DMSO. Cells were washed three times with pre-warmed 37° C Hanks’ balanced salt solution, and then opti-MEM (#11058021, Thermo Fisher Scientific, USA) culture medium was added. MitoSOX Green was added, and cells were incubated for 20 min. The mitochondrial ROS level was observed under a fluorescent microscope (Zeiss, Germany).

### The detection of intracellular ATP

After culturing for 24 hours, the culture medium was removed and 200 μL ATP lysis buffer was added to lyse the cells. Cells were then centrifuged at 4° C at 15,000 ×g for 2 min and the supernatant was collected. The ATP content of each group was calculated based on the standard curve.

### The measurement of the 8-OHdG level

The level of 8-OHdG was determined using the commercial 8-OHdG kit (#P-6003-48, EPIGENTEK, USA), following the instructions provided by the manufacturer. The binding solution was added to wells to bind DNAs, followed by the addition of capture and detector antibodies. The absorbance was detected at 450 nm after adding the developer solution.

### Western blotting assay

Total protein was extracted from cells using radioimmunoprecipitation assay (RIPA) lysis buffer. The protein concentration was measured using the bicinchoninic acid (BCA) kit (#D8284, Sigma-Aldrich, USA). Subsequently, the protein was separated by gel electrophoresis and transferred to a polyvinylidene fluoride (PVDF) membrane, which was then blocked. The membrane was incubated with primary antibodies against γH2AX (#A700-053, 1:1000, Thermo Fisher Scientific, USA), p53 (#9282, 1:1000, Cell Signaling Technology), p21 (#64016, 1:2000, Cell Signaling Technology), SIRT1 (#2028, 1:1000, Cell Signaling Technology), and β-actin (#4967, 1:5000, Cell Signaling Technology, USA) overnight, washed, and then incubated with a secondary antibody (#7074 or #7076, 1:2000, Cell Signaling Technology, USA) for 120 min. The membrane was then cultured with enhanced chemiluminescence (ECL) (#32106, Thermo Fisher Scientific, USA) solution, and images were captured using a gel imaging analysis system. The grayscale analysis was performed using ImageJ software.

### Tartrate-resistant acid phosphatase (TRAP)-silver staining assay for the detection of the telomerase activity

After collection, cells were added to pre-cooled lysis buffer and 0.5 μL 8-mercaptoethanol and incubated on ice for 30 min. Cells were then centrifuged at 4° C at 13,000 rpm for 30 min, and the cell fragments were removed. The supernatant was collected, and the protein concentration was measured using the BCA method. 2 μL of protein extract was added with the TRAP reaction solution, dNTPs, Taq-DNA polymerase, and primers, which were incubated at 23° C for 10 min. The CX primer was then added, mixed well, and PCR amplification was performed. 10 μL of PCR product was subjected to 12% non-denaturing polyacrylamide gel electrophoresis at 180 V for 1 hour. The gel was removed and fixed with 10% acetic acid for 30 min and soaked in 0.2 g/L sodium thiosulfate for 1 min. The gel was then washed three times with double-distilled water and stained with silver nitrate solution for 30 min and developed with toluidine blue solution for 10 min with slight shaking until a ladder-like DNA band appeared. The reaction was terminated by adding 10% acetic acid and results were analyzed using GelPro software.

### The senescence-associated-β-galactosidase (SA-β-Gal) assay

The supernatant was discarded, and cells were then fixed with 4% paraformaldehyde for 15 min. After fixation, cells were washed and stained with SA-β-Gal solution at 37° C overnight. Under a microscope, images were taken and the appearance of a blue color indicated positive staining of β-galactosidase. Five high-power fields were randomly selected, and the number of positive cells in 200 cells was counted to calculate the positive rate.

### Real-time (RT) PCR assay

1 mL Trizol (#T9424, Merck, USA) was added to cells, which were then lysed for 5 min. Total RNA was extracted and its concentration was detected using a NanoDrop 2000 spectrophotometer. 1 μg RNA was then reverse-transcribed into cDNA and subjected to real-time fluorescence quantitative PCR. The reaction system of 20 μL contained 10 μmol upstream primer, 10 μmol downstream primer, UltraSYBR Mixture, 40 ng cDNA template, and ddH_2_O. The Light Cycler 96 Real-Time PCR System was used for detection. β-actin was used as the negative control, and the data were calculated using the 2^-ΔΔCT^ formula.

### Statistical analysis

The experimental data were expressed as mean ± standard deviation and analyzed using SPSS18.0 statistical software. Multiple group comparisons were performed using a one-way analysis of variance. P<0.05 was considered a significant difference.

### Data availability statement

The data that support the findings of this study are available from the corresponding author upon reasonable request.

### Consent to publish

All the authors agreed to publish this article.
